# Study on the migration regularity of volatile flavor compounds during the roasting process of Tartary buckwheat tea based on HS-GC-IMS and chemometrics

**DOI:** 10.3389/fnut.2026.1777132

**Published:** 2026-07-02

**Authors:** Xianzhou Zhu, Li Zhang, Zou Dai, Ju Guan, Tianyang Wang

**Affiliations:** 1 Liangshan Branch of Sichuan Tobacco Company, Liangshan, Sichuan, China; 2 Xichang University, Xichang, Sichuan, China; 3 Key Laboratory of Culinary Science, Sichuan Tourism College, Chengdu, Sichuan, China

**Keywords:** chemometrics, flavor markers, GC-IMS, roasting process, Tartary buckwheat tea

## Abstract

Roasting is a critical processing step that strongly influences the chemical composition, volatile profile, and overall quality of Tartary buckwheat tea. This study investigated the dynamic changes in volatile flavor compounds during roasting using headspace-gas chromatography-ion mobility spectrometry (HS-GC-IMS) combined with chemometric analysis. Roasting significantly increased the contents of total flavonoids (2.29 g/100 g) and protein (15.67 g/100 g), while reducing the moisture content (5.77 g/100 g), particularly at stages X4-X6 (15–25 min). A total of 79 volatile compounds were identified by HS-GC-IMS, among which alcohols and ketones were predominant. Stages X3-X5 (10–20 min) were identified as the critical period for flavor accumulation. Partial least squares discriminant analysis (PLS-DA) identified 13 key discriminant compounds, including propyl acetate, hexyl butyrate, isoamyl formate, sec-butyl acetate, propanal, acrolein, 3-methyl-2-butenal, (E)-2-heptenal, methyl heptenone, 3-methyl-2-pentanone, 2-hexanone, sec-butanol, and 1-octene, as potential flavor markers. These findings clarify the roasting-dependent flavor evolution of Tartary buckwheat tea and provide a theoretical basis for process optimization to improve aroma quality.

## 1 Introduction

Tartary buckwheat (*Fagopyrum tataricum* (L.) Gaertn.), a medicinal and edible grain crop, is rich in nutrients and exhibits notable health-promoting properties (1). It is abundant in proteins, dietary fiber, vitamins, and other essential nutrients, and also contains bioactive compounds such as flavonoids, which are not typically found in other gramineous cereal crops (2, 3). Pharmacological animal studies and clinical observations have demonstrated that these bioactive components have significant effects in lowering blood glucose and lipid levels, as well as enhancing immunoregulatory functions (4, 5). With the rising prevalence of lifestyle-related diseases such as hyperglycemia, hyperlipidemia, and hypertension, processed products derived from Tartary buckwheat have gained increasing popularity among consumers. Among these, Tartary buckwheat tea has become a mainstream product due to its high added value and simple production process. Roasting is the key processing step in Tartary buckwheat tea production and significantly influences the formation and transformation of volatile flavor compounds through pathways such as the Maillard reaction and caramelization, thereby determining product quality (6). Therefore, a systematic investigation of the migration regularity changes in volatile components during Tartary buckwheat tea roasting can provide a theoretical foundation for process optimization and offer valuable insights for quality monitoring in industrial production.

Roasting, a critical thermal processing technique, is widely employed in the processing of grains such as barley, oats, and quinoa to enhance their physicochemical properties and flavor quality. McGorrin et al. (7) demonstrated that roasting significantly promotes flavor development in oatmeal, identifying over 110 compounds, including the key marker 2-methyl-3-furanthiol. Similarly, Sharma et al. (8) reported that dry roasting significantly improves the antioxidant activity of quinoa, suggesting that appropriate thermal treatment can simultaneously optimize both nutritional and functional characteristics of grains. During high-temperature roasting, precursors such as reducing sugars and amino acids undergo the Maillard reaction, caramelization, and Strecker degradation to generate key flavor compounds-such as pyrazines and furans-that impart a unique roasted aroma (9). In the specific case of Tartary buckwheat tea, the synergistic interaction of hexanal, pyrazines, and furans generated by these reactions is considered crucial for its distinctive flavor profile. For Tartary buckwheat, roasting is the critical step determining product quality; it not only governs the formation and transformation of volatile compounds via the aforementioned chemical pathways but also induces changes in bioactive constituents (10). However, most existing studies focus on characterizing the flavor of the final product, with relatively little research addressing the migration regularity of components during the roasting process.

Conventional gas chromatography-mass spectrometry (GC-MS) has limitations in the rapid detection and migration tracking of trace volatile compounds within complex matrices and is inadequate for resolving nonlinear interactions among multiple components (6). Due to its high sensitivity, minimal requirement for complex pretreatment, and ability to generate visual fingerprint maps, gas chromatography-ion mobility spectrometry (GC-IMS) has emerged as a powerful tool for the migration analysis of food flavors (11, 12). When combined with chemometric techniques such as principal component analysis (PCA) and partial least squares discriminant analysis (PLS-DA), GC-IMS enables deeper exploration of intrinsic relationships, thereby revealing key flavor markers and their migration pathways (13). Previous studies have demonstrated that the characteristic flavor of Tartary buckwheat tea results from the synergistic effects of hexanal, pyrazines, and furan compounds (9). In addition, GC-IMS has been applied to process monitoring in coffee roasting and tea fermentation, among others. Its rapid response and high resolution capabilities enable it to track the dynamic changes of volatile components in complex reaction systems in real time (14). Chemometric approaches like PLS-DA effectively extract key differential compounds through dimensionality reduction and pattern recognition, facilitating the establishment of process-component association models (15).

This study systematically elucidated the migration evolution of volatile compounds during the roasting process of Tartary buckwheat tea by combining gas chromatography-ion mobility spectrometry with chemometric analysis. It also identified key volatile markers associated with roasting. The aim of this research was to clarify the time-dependent changes in flavor characteristics of Tartary buckwheat tea during roasting, thereby providing a theoretical basis for quality control and standardized production.

## 2 Materials and methods

### 2.1 Preparation of buckwheat tea samples

Fresh, intact, and plump seeds of Tartary buckwheat (*Fagopyrum tataricum* (L.) Gaertn.) were obtained from Chengdu Food Technology Co., Ltd. (Chengdu, China). The roasting process was conducted according to the method described by Sui et al. (16) with slight modifications, establishing a final roasting duration of 25 min. Damaged grains and impurities were removed, after which the samples were rinsed with water and drained; subsequently, the seeds were wrapped in gauze, soaked in distilled water at 25 ± 2 °C for 2 h, and surface-dried to ensure a consistent initial moisture state prior to roasting. Roasting was performed in an electric oven (DZF-6020 Yetuo Shanghai, China) equipped with automatic temperature control, preheated for at least 30 min to ensure stability. Drained samples were spread in a layer on stainless steel trays and roasted at 120 ± 5 °C for 25 min, with the temperature maintained by an internal controller and monitored via a thermometer. To investigate the migration evolution of components, samples were collected at 5-min intervals and labeled as follows: X1 (0 min, unroasted control), X2 (5 min), X3 (10 min), X4 (15 min), X5 (20 min), and X6 (25 min). The sampling interval was established based on preliminary experiments, and the oven door remained open for ≤ 30 s during each collection to minimize temperature fluctuations. Samples were immediately cooled to ambient temperature to terminate thermal reactions, packed in sealed polyethylene bags, and stored at room temperature prior to analysis. All sample preparation procedures were performed in triplicate under identical conditions.

### 2.2 Determination of total flavonoids content

The total flavonoid content was determined using the colorimetric method described by Guan et al. (17). Briefly, 0.05 g of sample powder was mixed with 25 ml of 70% ethanol and subjected to ultrasonic extraction at room temperature (25 ± 3 °C) for 30 min. The mixture was subsequently centrifuged at 12,000 × g and filtered to obtain a clarified sample solution. Rutin (Shanghai Yuanye Bio-Technology Co., Ltd., Shanghai, China) was used as the standard to construct a calibration curve (*y* = 0.3649x + 0.0968, *R*^2^ = 0.9993) using a 0.028 mg/mL stock solution, with a linear range of 0.02–0.20 mg/ml. An aliquot of 1.00 ml of the sample solution was mixed with 1.00 ml of aqueous sodium nitrite (NaNO_2_) solution. After incubation for 6 min, 1.00 ml of 10% (w/v) aluminum nitrate (Al(NO_3_)_3_) solution was added, and the mixture was allowed to react for another 6 min. Subsequently, 5.00 ml of 10% (w/v) sodium hydroxide (NaOH) was added, and the mixture was diluted to a final volume of 25.00 ml with 70% ethanol. After 15 min, the absorbance was measured at 510 nm, and the total flavonoid content was calculated based on the rutin standard. In this assay, NaNO_2_ provides nitrite ions that react with flavonoid phenolic groups to promote chromophore formation and improve color development, while Al(NO_3_)_3_ supplies Al^3+^ to chelate flavonoids and form a stable colored complex. Total flavonoid content are expressed on a dry weight basis (DW).

### 2.3 Determination of energy and nutritional components

Using the food analysis mode of an energy analyzer (CA-HM, WP, Tokyo, Japan), the energy (kcal/100 g) and proximate components, including protein, fat, carbohydrate, and moisture (g/100 g), were quantitatively determined for crushed Tartary buckwheat samples according to Cai et al. (18). Briefly, approximately 100.00 g of each sample was evenly spread on a sample tray and scanned by near-infrared irradiation (1,100–2,200 nm). All results reported in Table 1 are expressed on a as-is weight basis. To ensure stable instrument performance, the analyzer was preheated for at least 30 min before testing, and each sample was measured five times. Energy, protein, fat content, and carbohydrate are expressed on a dry weight basis (DW), while only moisture content is expressed on a fresh weight basis (FW).

**Table 1:** The results of total flavonoid content and nutritional components during the roasting process of Tartary buckwheat tea.

Index	Tartary buckwheat tea sample					
	X1	X2	X3	X4	X5	X6
Total flavonoid content (g/100 g)	1.27 ± 0.09^d^	1.83 ± 0.06^c^	1.95 ± 0.032^bc^	2.15 ± 0.04^ab^	2.30 ± 0.12^a^	2.29 ± 0.11^a^
Energy (kcal/100 g)	3.44 ± 0.03^c^	3.56 ± 0.03^b^	3.64 ± 0.05^a^	3.62 ± 0.02^ab^	3.61 ± 0.01^ab^	3.62 ± 0.00^ab^
Protein (g/100 g)	14.63 ± 0.15^c^	15.07 ± 0.12^b^	15.27 ± 0.23^ab^	15.43 ± 0.22^ab^	15.54 ± 0.03^a^	15.67 ± 1.06^a^
Fat content (g/100 g)	7.27 ± 0.31^a^	6.83 ± 0.06^a^	6.73 ± 0.55^a^	6.53 ± 0.29^ab^	5.83 ± 0.06^bc^	5.50 ± 0.36^c^
Carbohydrate (g/100 g)	56.13 ± 0.15^b^	58.07 ± 1.07^a^	58.17 ± 1.91^a^	60.40 ± 0.10^a^	59.77 ± 0.15^a^	59.60 ± 0.20^a^
Moisture content (g/100 g)	36.77 ± 1.58^a^	23.97 ± 2.63^b^	16.07 ± 0.64^c^	12.33 ± 0.49^d^	7.73 ± 0.21^e^	5.77 ± 0.58^e^

Values are presented as mean ± SD (n = 5). Different lowercase letters within the same row indicate significant differences (p < 0.05) based on one-way ANOVA followed by Tukey's post hoc test. Total flavonoid content, energy, protein, fat content, and carbohydrate are expressed on a dry weight basis (DW), while only moisture content is expressed on a fresh weight basis (FW). Total flavonoid content is expressed as g/100 g, converted from mg/g using the relationship 1 mg/g = 0.10 g/100 g.

### 2.4 Determination of volatile components

Accurately weigh 2.00 g of Tartary buckwheat tea and place it into a 20.00 ml headspace vial for subsequent analysis. Prepare three parallel replicates for each sample (19). Headspace sampling conditions: incubate the sample at 60 °C for 10 min. An autosampler precisely introduces 1.50 ml of headspace into the syringe; injections are performed in splitless mode, with the syringe temperature maintained at 85 °C. The drift gas flow rate is set to 150 ml/min. GC conditions (20): use an MXT-WAX column (30 m × 0.53 mm, 1.00 μm) with the column temperature set at 60 °C. High-purity N_2_ (≥99.99%) serves as both the carrier and drift gases. The total analysis time is 35 min. Flow program: initial flow rate of 2 ml/min for 5 min; then 10 ml/min for 10 min; followed by 15 ml/min for 5 min; then 50 ml/min for 10 min; finally, increase the flow to 100 ml/min and hold for 5 min. Calculations were performed using C_4_-C_9_ n-ketones as external standards, and compound identification was confirmed by comparing retention indices and ion drift times with reference values in the IMS library (19).

### 2.5 Statistical analysis

Processing and analysis of GC-IMS data were conducted using the instrument's built-in Laboratory Analytical Viewer (LAV) and Gallery Plot. GC-IMS data were annotated in LAV and matched against the NIST and IMS databases. Subsequently, Gallery Plot was used to generate fingerprint plots of the samples. PLS-DA was performed on the HS-GC-IMS results using SIMCA 14.0, and the VIP value for each compound was calculated. Statistical significance was assessed using analysis of variance (ANOVA), followed by Tukey's HSD test for multiple comparisons, implemented in R software (version 4.4.3), with a significance threshold set at *p* < 0.05. Significance annotations were generated using the agricolae package. Bar plots charts were produced using Origin 2025 software (OriginLab, Northampton, MA, USA).

## 3 Results

### 3.1 Analysis of total flavonoid content and nutritional components during the roasting process of buckwheat tea

Total flavonoid content and nutritional components play crucial roles in the functional properties and flavor quality of buckwheat tea. A previous study reported that roasting significantly enhances the release efficiency of flavonoids in buckwheat tea and promotes the enrichment of nutrients such as proteins and lipids (21). As shown in Table 1, total flavonoids increased significantly with roasting time, rising from 1.27 ± 0.09 g/100 g in X1 to a peak of 2.30 ± 0.12 g/100 g in X5, followed by a slight decrease at X6 while remaining high (2.29 ± 0.11 g/100 g). This indicates that roasting favors flavonoid release particularly during the mid-to-late stages possibly due to cellular structural disruption and the conversion of bound flavonoids to free forms. The energy value increased slightly during roasting, rising from 3.44 ± 0.03 kcal in X1 to 3.64 ± 0.05 kcal in X3, remaining between 3.61 and 3.62 kcal. Although the magnitude of change was small, protein content exhibited a continuous upward trend, increasing from 14.63 ± 0.15 g/100 g in the unroasted sample (X1) to 15.67 ± 0.06 g/100 g in X6. Moreover, no significant difference was observed between X5 and X6 (*p* > 0.05), indicating that protein content tended to stabilize in the late roasting stage. In contrast, fat content decreased continuously, dropping from 7.27 ± 0.31 g/100 g in X1 to 5.50 ± 0.36 g/100 g in X6, with a significant reduction (*p* < 0.05), which attributable to lipid volatilization and thermal degradation at elevated temperatures (22).

Carbohydrate content remained relatively high throughout roasting, measuring 56.13 ± 0.15 g/100 g in X1 and reaching a maximum of 60.40 ± 0.10 g/100 g in X4, followed by a slight decrease with no significant overall change, indicating relative stability. Moisture content decreased markedly during roasting and represented the most pronounced change among all measured indices. Moisture content was 36.77 ± 1.58 g/100 g in X1 and gradually decreased to 5.77 ± 0.58 g/100 g in X6 after roasting. Notably, the largest reduction in moisture occurred during the first three stages (X1-X3), indicating that the primary dehydration effect of roasting was concentrated in the initial phase. In summary, roasting significantly affected the nutritional composition of Tartary buckwheat tea. Particularly in the mid-to-late stages (X4–X6), total flavonoids (2.29 ± 0.11 g/100 g) and protein content (15.67 ± 0.06 g/100 g) reached their highest levels, whereas moisture decreased markedly to 5.77 ± 0.58 g/100 g.

### 3.2 Analysis of HS-GC-IMS Spectra during the roasting process of buckwheat tea

**Figure 1:**
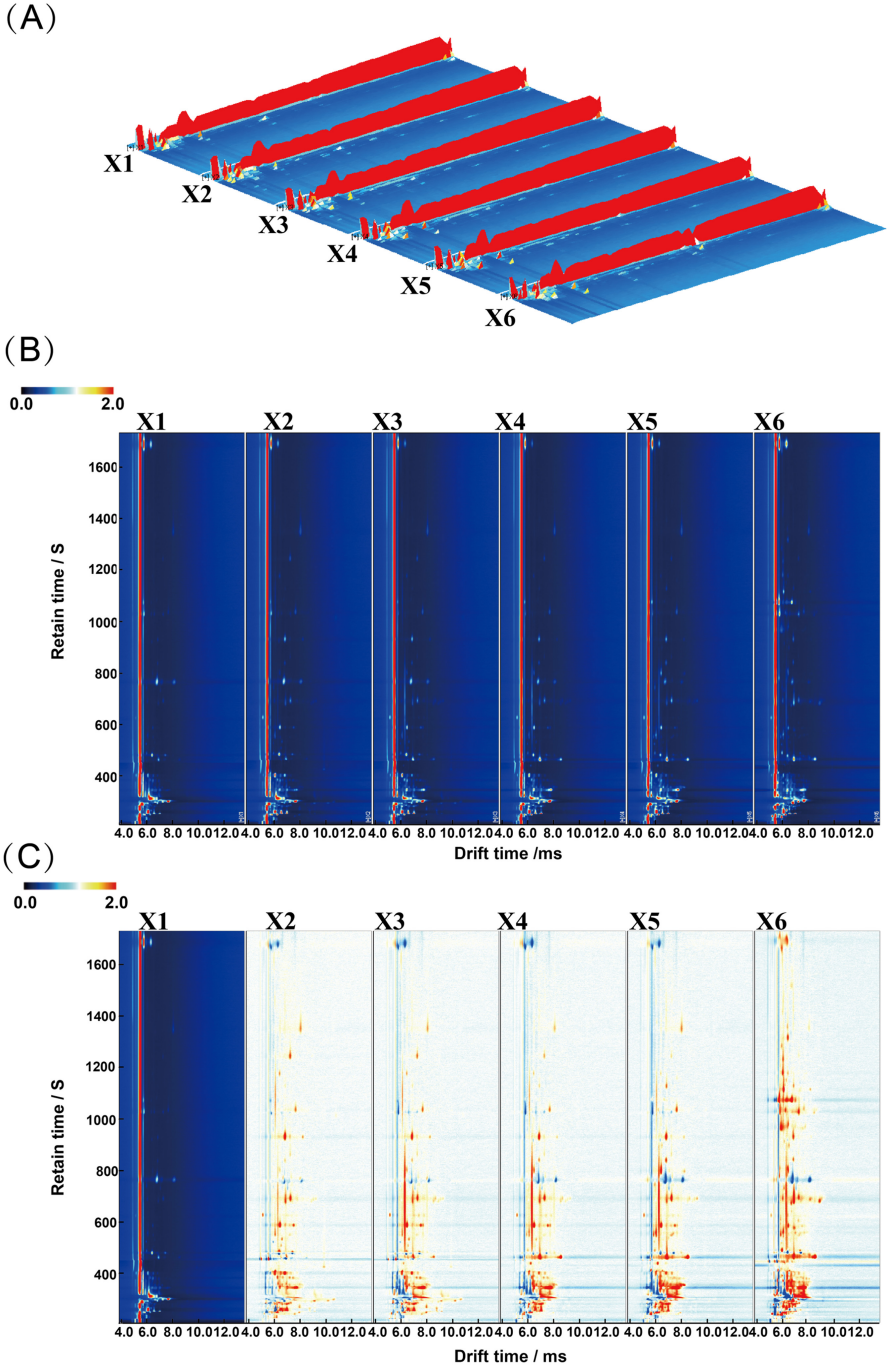
HS-GC-IMS spectra of the roasting process of Tartary buckwheat tea **(A)** Three-dimensional spectra, **(B)** Two-dimensional spectra, **(C)** Subtracting spectra. The background is light in color. The darker vertical lines on the left represent the peaks of reactive ions. Each point corresponds to a volatile compound, and the depth of the color indicates the peak intensities of the substance.

To further investigate the migration regularity characteristics of volatile components in Tartary buckwheat tea during roasting, HS-GC-IMS was used to systematically analyze samples from six roasting stages (X1–X6), and the corresponding spectra are shown in Figure 1. The three-dimensional plot in Figure 1A reveals the migration trend of the overall peak features of volatile compounds across different stages. The results showed that, at the early roasting stages (X1 and X2), the peak areas were small, indicating limited types of volatile components. With prolonged roasting, the number and intensity of signal peaks continuously increased from X3 to X5, indicating continuous accumulation of aroma-related substances and gradual enhancement of flavor release. By X6, the signals tended to stabilize, implying that the accumulation of flavor substances reached a steady state. This trend was consistent with the changes observed for nutritional factors such as total flavonoids and protein. The two-dimensional plot in Figure 1B further shows differences in signal responses among samples in terms of retention time and drift time. Although the overall structures of the spectra were broadly similar among roasting stages, pronounced differences were observed in peak intensity and signal positions. Particularly in the region of 400–800 s retention time and 5–9 ms drift time, numerous new peaks appeared in X3–X5, indicating that this stage is a key window for rapid release of flavor components. The differential plot in Figure 1C, using X1 as the reference, showed the changes in compound distribution for the other samples relative to X1. In the plot, red indicates regions with increased signal intensity, whereas blue represents attenuated signals. These results indicate that the red signals in samples X2–X6 gradually intensified during roasting, with the most pronounced increase occurring from X4 to X5, possibly due to the concentrated release of multiple newly formed volatiles at this stage. By X6, although further signal enhancement was still observed, the overall growth rate markedly slowed, suggesting that the flavor characteristics gradually became stable.

**Figure 2:**
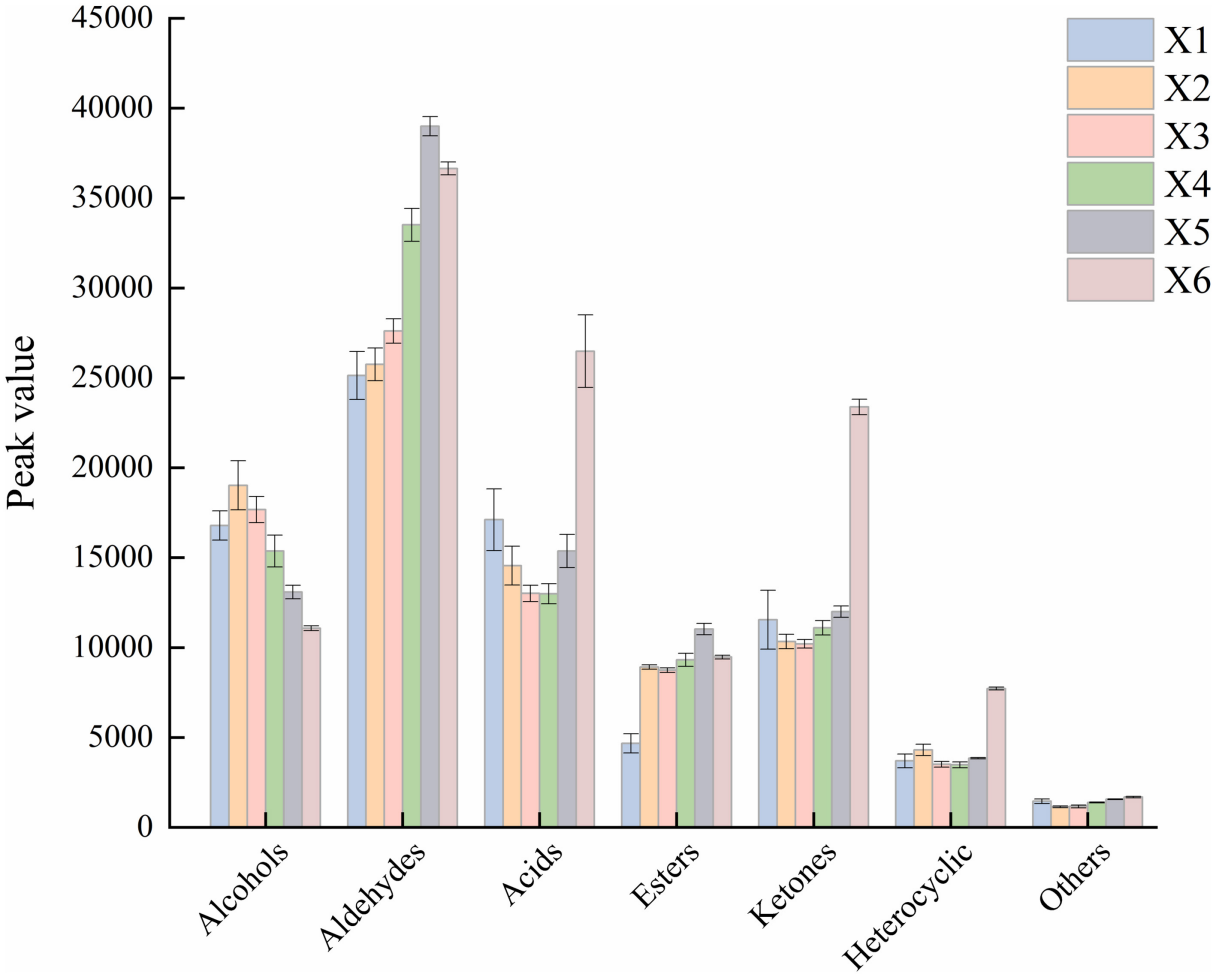
Categories of volatile compounds in Tartary buckwheat tea during the roasting process.

Compounds were identified by comparing their calculated RI and ion drift times with entries in the HS-GC-IMS database (20, 23). Notably, GC-IMS can precisely detect and identify certain volatile monomers and dimers, which is challenging to accomplish with conventional GC-MS (22). In total, 79 volatile compounds were detected and identified across the six samples, with detailed results presented in Table 2. Among these, alcohols and ketones constituted the largest proportions in terms of compound number. Regarding peak intensities of compound classes, the bar chart in Figure 2 illustrates the peak intensities of each class at different roasting stages. The peak intensities of aldehydes were low at X1, increased rapidly with roasting time, and peaked notably at X5, indicating significant aldehyde accumulation during roasting. At X6, the peak intensities decreased slightly but remained higher than the initial level, suggesting that aldehyde accumulation tended to stabilize in the late roasting stage. The peak intensities of acids changed relatively steadily, although a more pronounced increase was observed at X4, particularly due to the accumulation of some short-chain fatty acids. This may be related to fatty acid decomposition and esterification reactions during roasting, implying that acid formation is closely associated with roasting time. In contrast, ketone peak intensities increased continuously from X2 to X5, reaching the highest level at X4. Ketones are important products formed when carbohydrates undergo Maillard reactions or other thermal degradation processes during roasting, their increase likely reflects carbohydrate degradation and flavor compound formation during roasting (24, 25). The peak intensities of heterocyclic and other compounds were relatively low but also exhibited a gradual upward trend with extended roasting time. Notably, at X6, the peak intensities of heterocyclic compounds were relatively pronounced, which may be closely related to complex chemical reactions during roasting, such as amino acid pyrolysis.

**Table 2:** Volatile compounds identified during the roasting process of Tartary buckwheat tea.

Class	Compounds	Cas	Formula	Retention index	Retention time	Tartary buckwheat tea sample						Aroma description
						x1	x2	x3	x4	x5	x6	
Alcohols	1-butanol, 3-methyl-m	123-51-3	C_5_H_12_O	1216.90	764.38	5.35 × 10^3^ ± 1.56 × 10^2a^	4.65 × 10^3^ ± 4.08 × 10^ab^	4.23 × 10^3^ ± 3.28 × 10^2bc^	3.54 × 10^3^ ± 3.67 × 10^2c^	2.52 × 10^3^ ± 1.62 × 10^1d^	2.30 × 10^3^ ± 8.32 × 10^1d^	–
	1-butanol, 3-methyl-d	123-51-3	C_5_H_12_O	1217.00	764.74	2.24 × 10^3^ ± 1.30 × 10^2a^	1.99 × 10^3^ ± 1.98 × 10^2a^	1.86 × 10^3^ ± 1.93 × 10^2ab^	1.48 × 10^3^ ± 1.80 × 10^2b^	1.01 × 10^3^ ± 7.01^c^	8.62 × 10^2^ ± 1.96 × 10^1d^	–
	1-pentanol-m	71-41-0	C_5_H_12_O	1268.20	931.04	4.29 × 10^2^ ± 1.78 × 10^2c^	1.39 × 10^3^ ± 7.42 × 10^1a^	1.32 × 10^3^ ± 8.60 × 10^1a^	1.21 × 10^3^ ± 3.19 × 10^1a^	1.19 × 10^3^ ± 2.43 × 10^a^	7.74 × 10^2^ ± 1.63 × 10^1b^	–
	1-propanol	71-23-8	C_3_H_8_O	1045.40	403.49	1.66 × 10^3^ ± 2.98 × 10^2b^	3.18 × 10^3^ ± 2.88 × 10^2a^	2.96 × 10^3^ ± 1.69 × 10^2a^	2.55 × 10^3^ ± 2.14 × 10^2a^	2.47 × 10^3^ ± 3.28 × 10^2a^	1.26 × 10^3^ ± 3.80 × 10^1^ ^c^	–
	1-propanol, 2-methyl-m	78-83-1	C_4_H_10_O	1103.70	481.66	2.21 × 10^3^ ± 1.90 × 10^2a^	2.19 × 10^3^ ± 1.86 × 10^2a^	2.17 × 10^3^ ± 1.62 × 10^2a^	1.80 × 10^3^ ± 1.37 × 10^2a^	1.39 × 10^3^ ± 1.77 × 10^1b^	1.31 × 10^3^ ± 3.32 × 10^1b^	–
	1-propanol, 2-methyl-d	78-83-1	C_4_H_10_O	1103.70	481.66	5.00 × 10^2^ ± 1.10 × 10^2a^	4.18 × 10^2^ ± 6.08 × 10^1ab^	4.33 × 10^2^ ± 8.85 × 10^1ab^	3.10 × 10^2^ ± 5.24 × 10^1^ ^b^	1.95 × 10^2^ ± 9.82 ^c^	1.67 × 10^2^ ± 3.55 ^c^	–
	2-propanol	67-63-0	C_3_H_8_O	965.60	325.70	1.77 × 10^3^ ± 6.83 × 10^1a^	1.04 × 10^3^ ± 1.36 × 10^2b^	6.87 × 10^2^ ± 2.91 × 10^1c^	7.51 × 10^2^ ± 3.86 × 10^1c^	9.17 × 10^2^ ± 5.43 × 10^1b^	1.07 × 10^3^ ± 3.49 × 10^1b^	–
	2-propanol	67-63-0	C_3_H_8_O	917.60	298.12	7.96 × 10^2^ ± 2.74 × 10^1a^	6.52 × 10^2^ ± 2.57 × 10^1b^	5.33 × 10^2^ ± 1.36 × 10^1c^	5.30 × 10^2^ ± 1.52 × 10^1c^	5.47 × 10^2^ ± 1.78 × 10^1c^	4.16 × 10^2^ ± 1.90 × 10^1d^	–
	2- butanol	78-92-2	C_4_H_10_O	1030.10	384.73	1.82 × 10^2^ ± 2.11 × 10^1d^	4.48 × 10^2^ ± 1.85 × 10^1b^	5.36 × 10^2^ ± 3.24 × 10^a^	4.70 × 10^2^ ± 1.80 × 10^1b^	3.37 × 10^2^ ± 1.88^c^	3.64 × 10^2^ ± 2.55^c^	–
	1 -hexanol	111-27-3	C_6_H_14_O	1369.70	1242.24	3.46 × 10^2^ ± 5.40 × 10^1d^	7.02 × 10^2^ ± 5.11 × 10^1a^	6.23 × 10^2^ ± 2.80 × 10^1ab^	4.98 × 10^2^ ± 4.86 × 10^1bc^	3.99 × 10^2^ ± 1.75 × 10^1cd^	3.18 × 10^2^ ± 7.32^d^	–
	1-pentanol-d	71-41-0	C_5_H_12_O	1268.40	931.77	9.38 × 10^1^ ± 6.33^c^	2.13 × 10^2^ ± 7.72^a^	1.91 × 10^2^ ± 1.45 × 10^1a^	1.76 × 10^2^ ± 1.31 × 10^1a^	1.74 × 10^2^ ± 2.21 × 10^1a^	1.18 × 10^2^ ± 1.42 × 10^1b^	–
	butanol	71-36-3	C_4_H_10_O	1151.10	589.81	5.82 × 10^1^ ± 3.56^d^	2.62 × 10^2^ ± 1.61 × 10^1ab^	3.07 × 10^2^ ± 2.42 × 10^1a^	2.90 × 10^2^ ± 2.36 × 10^1ab^	2.46 × 10^2^ ± 9.82^b^	1.01 × 10^2^ ± 8.77^c^	–
	1-penten-3-ol	616-25-1	C_5_H_10_O	1167.40	626.98	6.84 × 10^2^ ± 1.09 × 10^1c^	1.16 × 10^3^ ± 6.19 × 10^1a^	1.13 × 10^3^ ± 4.27 × 10^1a^	1.14 × 10^3^ ± 3.55 × 10^1a^	1.17 × 10^3^ ± 5.54^a^	9.41 × 10^2^ ± 1.64 × 10^1b^	–
	2-pentanol	6032-29-7	C_5_H_12_O	1125.70	531.94	1.33 × 10^2^ ± 5.41^c^	2.69 × 10^2^ ± 1.18 × 10^1a^	2.49 × 10^2^ ± 1.63 × 10^1ab^	2.17 × 10^2^ ± 1.05^b^	1.50 × 10^2^ ± 1.26^c^	1.45 × 10^2^ ± 1.07 × 10^1c^	–
	2-pentanol	6032-29-7	C_5_H_12_O	1115.80	509.41	1.20 × 10^2^ ± 6.17^d^	1.98 × 10^2^ ± 1.42 × 10^1a^	2.00 × 10^2^ ± 1.52 × 10^1a^	1.76 × 10^2^ ± 5.70^ab^	1.49 × 10^2^ ± 1.06 × 10^1bc^	1.32 × 10^2^ ± 1.10 × 10^1cd^	–
	(*e*)-2-hexen-1-ol	928-95-0	C_6_H_12_O	1396.00	1321.33	2.17 × 10^2^ ± 3.11 × 10^1b^	2.45 × 10^2^ ± 1.30 × 10^1b^	2.31 × 10^2^ ± 2.19 × 10^1b^	2.25 × 10^2^ ± 7.96^b^	2.27 × 10^2^ ± 1.44 × 10^1b^	8.10 × 10^2^ ± 8.69 × 10^1a^	–
Aldehydes	3-methyl-2-butenal^*^	107-86-8	C_5_H_8_O	1210.40	742.97	3.97 × v ± 2.33 × 10^1bc^	3.43 × 10^2^ ± 1.45 × 10^1d^	3.62 × 10^2^ ± 7.14^cd^	4.41 × 10^2^ ± 2.63 × 10^1b^	5.77v10^2^ ± 2.09^a^	4.31 × 10^2^ ± 2.10 × 10^1b^	Fresh apple peel
	heptanal	111-71-7	C_7_H_14_O	1194.00	689.62	3.90 × 10^2^ ± 1.00 × 10^1d^	1.09 × 10^3^ ± 2.09 × 10^1b^	1.09 × 10^3^ ± 8.60 × 10^1b^	1.15 × 10^3^ ± 1.05 × 10^1b^	1.50 × 10^3^ ± 5.24 × 10^1a^	9.33 × 10^2^ ± 1.60 × 10^1c^	–
	(*e*)-2-pentenal	1576-87-0	C_5_H_8_O	1137.60	559.09	2.60 × 10^2^ ± 3.41 × 10^1c^	4.61 × 10^2^ ± 8.79 × 10^1b^	5.28 × 10^2^ ± 2.19 × 10^1b^	5.76 × 10^2^ ± 5.24 × 10^1b^	9.25 × 10^2^ ± 1.17 × 10^2a^	8.45 × 10^2^ ± 3.94 × 10^1a^	–
	1-hexanal	66-25-1	C_6_H_12_O	1096.20	465.37	2.21 × 10^3^ ± 9.90 × 10^2ab^	1.62 × 10^3^ ± 9.04 × 10^2b^	2.65 × 10^3^ ± 4.00 × 10^2ab^	3.50 × 10^3^ ± 2.86 × 10^2ab^	4.14 × 10^3^ ± 9.74 × 10^1a^	3.54 × 10^3^ ± 4.04 × 10^1ab^	–
	butanal-m	123-72-8	C_4_H_8_O	851.40	260.10	1.40 × 10^4^ ± 5.30 × 10^2c^	9.97 × 10^3^ ± 2.59 × 10^2d^	1.04 × 10^4^ ± 2.84 × 10^2d^	1.35 × 10^4^ ± 1.77 × 10^2c^	1.56 × 10^4^ ± 2.76 × 10^2b^	1.90 × 10^4^ ± 3.31 × 10^2a^	–
	butanal-d	123-72-8	C_4_H_8_O	841.00	254.10	4.93 × 10^2^ ± 6.81 × 10^1e^	1.76 × 10^3^ ± 5.31 × 10^1a^	1.51 × 10^3^ ± 8.14 × 10^1b^	1.52 × 10^3^ ± 7.69 × 10^1b^	1.33 × 10^3^ ± 3.04 × 10^1c^	9.45 × 10^2^ ± 3.16 × 10^1d^	–
	2-methylbutanal-m	96-17-3	C_5_H_10_O	895.20	285.25	7.8 × 8v10^1^ ± 5.63^e^	1.77 × 10^2^ ± 1.74^d^	2.03 × 10^2^ ± 7.95^c^	2.81 × 10^2^ ± 3.05^b^	3.28 × 10^2^ ± 1.67^a^	1.92 × 10^2^ ± 6.58^c^	–
	2-methylbutanal-d	96-17-3	C_5_H_10_O	924.00	301.82	4.60 × 10^3^ ± 3.91 × 10^2c^	6.57 × 10^3^ ± 2.05 × 10^2b^	6.98 × 10^3^ ± × 1.01 × 10^2b^	7.81 × 10^3^ ± 2.50 × 10^2a^	7.99 × 10^3^ ± 8.14 × 10^1a^	6.73 × 10^3^ ± 1.15 × 10^2b^	–
	(*e*)-2-heptenal^*^	18829-55-5	C_7_H_12_O	1331.50	1127.73	1.90 × 10^2^ ± 6.13 × 10^1bc^	1.51 × 10^2^ ± 1.40 × 10^1c^	1.80 × 10^2^ ± 2.66 × 10^1bc^	2.15 × 10^2^ ± 2.47 × 10^1abc^	3.57 × 10^2^ ± 2.49 × 10^1a^	2.65 × 10^2^ ± 4.36^ab^	Nuts and cherries
	Acrolein^*^	107-02-8	C_3_H_4_O	835.20	250.78	1.70 × 10^3^ ± 7.75 × 10^1b^	2.07 × 10^3^ ± 5.66 × 10^1a^	1.71 × 10^3^ ± 2.82 × 10^1b^	1.77 × 10^3^ ± 8.09 × 10^1b^	1.83 × 10^3^ ± 3.65 × 10^1b^	1.30 × 10^3^ ± 1.66 × 10^1c^	Almond and church
	2-methyl-2-propenal	78-85-3	C_4_H_6_O	893.50	284.27	1.21 × 10^2^ ± 2.66 × 10^1e^	1.79 × 10^2^ ± 1.01 × 10^1d^	1.95 × 10^2^ ± 1.20 × 10^1cd^	2.26 × 10^2^ ± 3.73^c^	2.88 × 10^2^ ± 5.24^b^	3.25 × 10^2^ ± 9.14^a^	–
	(*e*)-2-hexen-1-al	6728-26-3	C_6_H_10_O	1228.20	801.18	3.16 × 10^2^ ± 6.10 × 10^1e^	4.30 × 10^2^ ± 3.87 × 10^1de^	5.15 × 10^2^ ± 2.21 × 10^1cd^	7.22 × 10^2^ ± 1.23 × 10^1c^	1.44 × 10^3^ ± 7.03 × 10^1a^	1.04 × 10^3^ ± 1.47 × 10^2b^	–
	Pentanal	110-62-3	C_5_H_10_O	998.80	346.56	1.15 × 10^2^ ± 3.95 × 10^1f^	6.32 × 10^2^ ± 4.83 × 10^1e^	1.23 × 10^3^ ± 1.03 × 10^2c^	1.77 × 10^3^ ± 6.83 × 10^1b^	2.59 × 10^3^ ± 6.29 × 10^1a^	9.66 × 10^2^ ± 3.24 × 10^1d^	–
	Propanal^*^	123-38-6	C_3_H_6_O	802.80	232.13	2.28 × 10^2^ ± 3.57 × 10^1a^	3.01 × 10^2^ ± 1.12 × 10^2a^	7.32 × 10^1^ ± 1.13 × 10^1bc^	6.11 × 10^1^ ± 1.87^c^	6.99 × 10^1^ ± 1.33^bc^	8.92 × 10^1^ ± 3.70^b^	Yeasty, with a potato aroma
Acids	Acetic acid	64-19-7	C_2_H_4_O_2_	1518.40	1688.18	1.37 × 10^4^ ± 1.04 × 10^3b^	1.20 × 10^4^ ± 7.01 × 10^2bcd^	1.10 × 10^4^ ± 3.51 × 10^2cd^	1.09 × 10^4^ ± 3.70 × 10^2d^	1.25 × 10^4^ ± 5.56 × 10^2bc^	1.81 × 10^4^ ± 7.62 × 10^2a^	–
	Acetic acid	64-19-7	C_2_H_4_O_2_	1518.50	1688.42	3.43 × 10^3^ ± 6.69 × 10^2b^	2.51 × 10^3^ ± 3.79 × 10^2bc^	2.05 × 10^3^ ± 1.11 × 10^2c^	2.06 × 10^3^ ± 1.86 × 10^2c^	2.82 × 10^3^ ± 3.67 × 10^2bc^	8.36 × 10^3^ ± 1.26 × 10^3a^	–
Esters&&&	Butanoic acid, hexyl ester	2639-63-6	C_10_H_20_O_2_	1406.80	1353.71	9.22 × 10^2^ ± 4.44 × 10^1d^	1.69 × 10^3^ ± 1.61 × 10^2a^	1.42 × 10^3^ ± 1.65 × 10^2ab^	1.32 × 10^3^ ± 6.23 × 10^1bc^	1.65 × 10^3^ ± 4.34 × 10^1a^	1.14 × 10^3^ ± 2.56 × 10^1c^	–
	Ethyl heptanoate	106-30-9	C_9_H_18_O_2_	1301.30	1037.27	1.50 × 10^2^ ± 1.16 × 10^1d^	4.52 × 10^2^ ± 3.38 × 10^1b^	4.88 × 10^2^ ± 5.65 × 10^1b^	4.49 × 10^2^ ± 1.16 × 10^1b^	5.96 × 10^2^ ± 2.58 × 10^1a^	2.74 × 10^2^ ± 7.74^c^	–
	Ethyl (*e*)-2-butenoate	623-70-1	C_6_H_10_O_2_	1150.00	587.26	2.24 × 10^2^ ± 2.37 × 10^1e^	1.34 × 10^3^ ± 3.27 × 10^1bc^	1.46 × 10^3^ ± 5.82 × 10^1a^	1.42 × 10^3^ ± 2.76 × 10^1b^	1.31 × 10^3^ ± 9.79^c^	7.18 × 10^2^ ± 2.10 × 10^1d^	–
	Methyl pentanoate	624-24-8	C_6_H_12_O_2_	1094.60	463.50	4.65 × 10^2^ ± 3.29 × 10^2bc^	2.90 × 10^2^ ± 2.75 × 10^2c^	6.07 × 10^2^ ± 1.91 × 10^2bc^	1.25 × 10^3^ ± 3.63 × 10^2b^	2.06 × 10^3^ ± 2.06 × 10^2a^	1.79 × 10^3^ ± 3.95 × 10^1a^	–
	Methyl acetate	79-20-9	C_3_H_6_O_2_	842.00	254.65	1.03 × 10^3^ ± 8.87 × 10^1d^	2.26 × 10^3^ ± 2.66 × 10^1a^	1.96 × 10^3^ ± 5.15 × 10^b^	1.89 × 10^3^ ± 7.59 × 10^1b^	1.88 × 10^3^ ± 3.23 × 10^1b^	1.40 × 10^3^ ± 1.50 × 10^1c^	–
	Sec-butyl acetate^*^	105-46-4	C_6_H_12_O_2_	994.30	342.21	4.17 × 10^2^ ± 6.69 × 10^1bc^	3.96 × 10^2^ ± 2.06 × 10^1bc^	3.48 × 10^2^ ± 9.73^c^	3.87 × 10^2^ ± 2.67 × 10^1c^	4.29 × 10^2^ ± 2.35^b^	7.37 × 10^2^ ± 1.38 × 10^1a^	Banana
	Acetic acid 2-propyl ester	108-21-4	C_5_H_10_O_2_	863.70	267.17	2.83 × 10^2^ ± 1.14 × 10^1d^	2.25 × 10^2^ ± 1.89 × 10^1e^	1.70 × 10^2^ ± 2.70^f^	3.71 × 10^2^ ± 1.59 × 10^1c^	5.70 × 10^2^ ± 3.22 × 10^1b^	1.41 × 10^3^ ± 3.27 × 10^1a^	–
	Acetic acid ethyl ester-m	141-78-6	C_4_H_8_O_2_	892.40	283.66	1.96 × 10^2^ ± 2.83 × 10^1e^	3.82 × 10^2^ ± 3.50 × 10^1d^	5.58 × 10^2^ ± 1.29 × 10^1b^	5.90 × 10^2^ ± 9.72^ab^	6.24 × 10^2^ ± 3.09^a^	4.84 × 10^2^ ± 6.66^c^	–
	Acetic acid ethyl ester-d	141-78-6	C_4_H_8_O_2_	895.40	285.40	9.94 × 10^1^ ± 7.73 × 10^1a^	1.47 × 10^2^ ± 2.16 × 10^1a^	1.28 × 10^2^ ± 9.56^a^	1.05 × 10^2^ ± 3.08^a^	8.89 × 10^1^ ± 7.22^a^	1.65 × 10^2^ ± 1.70 × 10^1a^	–
	Isopropyl butanoate	638-11-9	C_7_H_14_O_2_	1045.20	403.19	1.74 × 10^2^ ± 6.29 × 10^1b^	8.20 × 10^2^ ± 2.09 × 10^2a^	6.59 × 10^2^ ± 9.55 × 10^1a^	4.51 × 10^2^ ± 9.47 × 10^1a^	4.49 × 10^2^ ± 1.35 × 10^2a^	1.59 × 10^2^ ± 9.46^b^	–
	3-Methylbutyl formate^*^	110-45-2	C_6_H_12_O_2_	1027.50	381.59	1.95 × 10^2^ ± 1.95 × 10^1a^	2.17 × 10^2^ ± 2.93 × 10^1a^	1.76 × 10^2^ ± 9.99^ab^	2.02 × 10^2^ ± 2.84 × 10^1a^	2.11 × 10^2^ ± 2.20 × 10^1a^	1.50 × 10^2^ ± 1.05 × 10^1b^	Wine and fatty aromas
	Sec-butyl acetate^*^	105-46-4	C_6_H_12_O_2_	1007.30	357.00	1.53 × 10^2^ ± 8.74^e^	2.88 × 10^2^ ± 7.35^d^	3.42 × 10^2^ ± 1.55 × 10^1c^	3.67 × 10^2^ ± 3.81^bc^	6.46 × 10^2^ ± 2.67 × 10^1a^	3.95 × 10^2^ ± 1.36 × 10^1b^	Fruity aroma
	Acetic acid propyl ester^*^	109-60-4	C_5_H_10_O_2_	977.20	332.37	2.83 × 10^2^ ± 5.44 × 10^1b^	2.80 × 10^2^ ± 9.62^b^	2.92 × 10^2^ ± 1.77 × 10^1ab^	3.84 × 10^2^ ± 3.27 × 10^1a^	3.83 × 10^2^ ± 1.39 × 10^1a^	3.93 × 10^2^ ± 1.29 × 10^1a^	Fruity, banana and honey
	Butanoicac id propyl ester	105-66-8	C_7_H_14_O_2_	1145.60	577.43	7.45 × 10^1^ ± 8.39^c^	1.25 × 10^2^ ± 1.77 × 10^1b^	1.39 × 10^2^ ± 1.92^b^	1.34 × 10^2^ ± 1.51 × 10^1b^	1.29 × 10^2^ ± 3.17^b^	2.64 × 10^2^ ± 1.04 × 10^1a^	
Ketones	2-butanone, 3-hydroxy	513-86-0	C_4_H_8_O_2_	1299.50	1031.90	3.84 × 10^3^ ± 7.70 × 10^2a^	2.22 × 10^3^ ± 2.65 × 10^2b^	1.53 × 10^3^ ± 7.31 × 10^1c^	1.69 × 10^3^ ± 7.46 × 10^1bc^	1.68 × 10^3^ ± 7.37 × 10^1c^	3.75 × 10^3^ ± 5.14 × 10^1a^	–
	1-hydroxy-2-propanone-m	116-09-6	C_3_H_6_O_2_	1314.20	1075.93	2.91 × 10^3^ ± 1.25 × 10^2b^	2.18 × 10^3^ ± 1.16 × 10^2c^	1.97 × 10^3^ ± 4.93 × 10^1d^	2.27 × 10^3^ ± 1.42 × 10^2c^	2.43 × 10^3^ ± 1.00 × 10^2c^	6.80 × 10^3^ ± 1.29 × 10^2a^	–
	2-octanone	111-13-7	C_8_H_16_O	1298.60	1029.16	4.37 × 10^2^ ± 2.32 × 10^2ab^	1.76 × 10^2^ ± 3.14 × 10^1bc^	1.36 × 10^2^ ± 8.30^c^	1.34 × 10^2^ ± 1.76 × 10^1c^	1.44 × 10^2^ ± 7.29^c^	7.90 × 10^2^ ± 1.03 × 10^1a^	–
	Cyclopentanone	120-92-3	C_5_H_8_O	1194.40	690.92	2.03 × 10^2^ ± 1.25 × 10^1b^	2.08 × 10^2^ ± 2.53^b^	2.07 × 10^2^ ± 1.48 × 10^1b^	2.04 × 10^2^ ± 9.09^b^	1.92 × 10^2^ ± 9.03^b^	4.99 × 10^2^ ± 1.35 × 10^1a^	–
	4-methyl-2-pentanone	108-10-1	C_6_H_12_O	1027.50	381.56	5.92 × 10^2^ ± 2.22 × 10^1c^	8.24 × 10^2^ ± 9.82 × 10^1b^	1.04 × 10^3^ ± 2.99 × 10^1a^	9.93 × 10^2^ ± 1.05 × 10^2ab^	1.12 × 10^3^ ± 1.10 × 10^2a^	9.67 × 10^2^ ± 2.99 × 10^1ab^	–
	2-hexanone ^*^	591-78-6	C_6_H_12_O	1043.10	400.68	4.38 × 10^2^ ± 9.38 × 10^1bc^	6.44 × 10^2^ ± 7.85 × 10^1a^	5.15 × 10^2^ ± 1.07 × 10^1ab^	5.61 × 10^2^ ± 3.37 × 10^1ab^	5.21 × 10^2^ ± 4.80 × 10^1ab^	3.65 × 10^2^ ± 1.65 × 10^1c^	Fruity, meaty, buttery
	2,3-butanedione	431-03-8	C_4_H_6_O_2_	997.90	345.57	7.08 × 10^2^ ± 8.10 × 10^1d^	1.29 × 10^3^ ± 9.37 × 10^1c^	1.89 × 10^3^ ± 3.99 × 10^1b^	2.05 × 10^3^ ± 3.82 × 10^1ab^	2.10 × 10^3^ ± 4.18 × 10^1a^	1.89 × 10^3^ ± 4.53 × 10^1b^	–
	1-penten-3-one	1629-58-9	C_5_H_8_O	1019.80	372.19	5.02 × 10^2^ ± 8.02 × 10^1a^	2.66 × 10^2^ ± 3.58 × 10^1b^	2.29 × 10^2^ ± 1.78 × 10^1b^	2.55 × 10^2^ ± 1.51 × 10^1b^	2.79 × 10^2^ ± 1.16 × 10^1b^	4.37 × 10^2^ ± 1.76 × 10^1a^	–
	Cyclohexanone	108-94-1	C_6_H_10_O	1298.60	1029.12	2.44 × 10^2^ ± 7.47 × 10^1b^	1.77 × 10^2^ ± 1.79 × 10^1c^	1.53 × 10^2^ ± 1.37 × 10^1c^	1.84 × 10^2^ ± 1.51 × 10^1c^	2.18 × 10^2^ ± 4.91^c^	4.98 × 10^2^ ± 2.93 × 10^1a^	–
	2-heptanone-m	110-43-0	C_7_H_14_O	1192.20	683.83	1.41 × 10^2^ ± 1.09^c^	4.13 × 10^2^ ± 5.04 × 10^1b^	4.21 × 10^2^ ± 3.03 × 10^1b^	4.34 × 10^2^ ± 4.12 × 10^1b^	4.19 × 10^2^ ± 2.71 × 10^1b^	1.11 × 10^3^ ± 5.96 × 10^1a^	–
	3-penten-2-one	625-33-2	C_5_H_8_O	1095.30	464.33	3.88 × 10^2^ ± 1.61 × 10^2ab^	2.55 × 10^2^ ± 1.06 × 10^2b^	3.60 × 10^2^ ± 4.91 × 10^1ab^	4.87 × 10^2^ ± 7.29 × 10^1ab^	6.05 × 10^2^ ± 2.98 × 10^1a^	6.03 × 10^2^ ± 6.54^a^	–
	2-methyl-2-hepten-6-one^*^	110-93-0	C_8_H_14_O	1348.90	1179.94	2.17 × 10^2^ ± 1.51 × 10^1d^	4.12 × 10^2^ ± 1.95 × 10^1a^	3.66 × 10^2^ ± 3.37 × 10^1ac^	3.17 × 10^2^ ± 2.26 × 10^1c^	3.02 × 10^2^ ± 8.13^c^	3.78 × 10^2^ ± 2.69 × 10^1b^	Banana and a taste similar to green beans
	2-heptanone-d	110-43-0	C_7_H_14_O	1191.10	681.19	7.51 × 10^1^ ± 4.09^c^	1.31 × 10^2^ ± 4.81^b^	1.28 × 10^2^ ± 1.37 × 10^1b^	1.35 × 10^2^ ± 1.12 × 10^1b^	1.46 × 10^2^ ± 1.08 × 10^1b^	5.24 × 10^2^ ± 4.27 × 10^1a^	–
	3-methyl-2-pentanone^*^	565-61-7	C_6_H_12_O	1008.50	358.50	2.97 × 10^1^ ± 3.17^e^	7.34 × 10^1^ ± 3.10^d^	1.05 × 10^2^ ± 1.40 × 10^1c^	1.52 × 10^2^ ± 9.41^b^	3.78 × 10^2^ ± 2.45^a^	1.02 × 10^2^ ± 9.93^c^	Fruity, with a mild herbal aroma.
	2-pentanone	107-87-9	C_5_H_10_O	1028.00	382.22	5.14 × 10^1^ ± 1.43^c^	1.23 × 10^2^ ± 2.69 × 10^1ab^	1.45 × 10^2^ ± 2.03 × 10^1a^	1.11 × 10^2^ ± 1.77 × 10^1ab^	9.78 × 10^1^ ± 7.42^ab^	8.18 × 10^1^ ± 4.62^b^	–
	2-propanone	67-64-1	C_3_H_6_O	831.50	248.61	4.34 × 10^1^ ± 5.69^d^	7.94 × 10^1^ ± 2.81^c^	7.44 × 10^1^ ± 4.66^c^	1.50 × 10^2^ ± 1.18 × 10^1b^	2.80 × 10^2^ ± 1.86 × 10^1a^	3.20 × 10^2^ ± 1.61 × 10^1a^	–
	2-hexanone^*^	591-78-6	C_6_H_12_O	1093.10	461.63	1.69 × 10^2^ ± 4.68 × 10^1b^	2.19 × 10^2^ ± 3.95 × 10^1b^	3.20 × 10^2^ ± 2.16 × 10^1a^	3.10 × 10^2^ ± 2.20 × 10^1a^	3.21 × 10^2^ ± 9.67^a^	3.74 × 10^2^ ± 1.74 × 10^1a^	Fruity, meaty, buttery aroma
	2-methyl-2-cyclopenten-1-one	1120-73-6	C_6_H_8_O	1366.30	1232.03	2.63 × 10^2^ ± 3.33 × 10^1b^	3.02 × 10^2^ ± × 1.78 × 10^1b^	3.05 × 10^2^ ± 1.85 × 10^1b^	2.79 × 10^2^ ± 1.71 × 10^1b^	2.97 × 10^2^ ± 1.99 × 10^1b^	7.67 × 10^2^ ± 2.92 × 10^1a^	–
	1-hydroxy-2-propanone-d	116-09-6	C_3_H_6_O_2_	1314.30	1076.34	2.06 × 10^2^ ± 1.92 × 10^1c^	2.46 × 10^2^ ± 1.40 × 10^1c^	2.09 × 10^2^ ± 6.73^c^	2.37 × 10^2^ ± 1.20 × 10^1c^	2.85 × 10^2^ ± 3.77 × 10^1b^	2.70 × 10^3^ ± 1.64 × 10^2a^	–
	1-octen-3-one	4312-99-6	C_8_H_14_O	1314.20	1075.95	5.29 × v ± 3.78^b^	5.70 × 10^1^ ± 3.02^b^	5.53 × 10^1^ ± 3.83^b^	6.19 × 10^1^ ± 5.17^b^	6.48 × 10^1^ ± 9.55^b^	2.10 × 10^2^ ± 2.65^a^	–
	2-pentanone	107-87-9	C_5_H_10_O	962.10	323.73	3.46 × 10^1^ ± 1.39^d^	3.51 × 10^1^ ± 3.97^d^	4.67 × 101 ± 7.93^d^	7.59 × 101 ± 6.7 × 0^c^	1.07 × 10^2^ ± 4.67^b^	2.22 × 10^2^ ± 1.75 × 101^a^	–
Heterocyclic	2-pentyl furan	3777-69-3	C_9_H_14_O	1240.10	839.70	2.37 × 10^2^ ± 3.83 × 101^c^	2.84 × 10^2^ ± 7.99^c^	2.97 × 10^2^ ± 2.76 × 101^c^	3.70 × 10^2^ ± 2.53 × 101^b^	5.72 × 10^2^ ± 1.31 × 101^a^	3.97 × 10^2^ ± 7.7 × 1^b^	–
	ethyl benzene	100-41-4	C_8_H_10_	1121.80	523.10	1.47 × 10^2^ ± 3.65 × 101^cd^	1.22 × 10^2^ ± 10^1^7.31^d^	1.53 × 10^2^ ± 8.73^bcd^	1.98 × 102 ± 2.44^ac^	2.69 × 102 ± 1.99 × 10^1a^	2.16 × 102 ± 5.07^b^	–
	triethylamine	121-44-8	C_6_H_15_N	805.80	233.84	1.52 × 10^3^ ± 3.78 × 10^1b^	1.60 × 10^3^ ± 1.93 × 102^b^	8.80 × 102 ± 2.66 × 101^cd^	7.86 × 102 ± 5.48 × 101^d^	9.97 × 102 ± 6.14^c^	3.11 × 10^3^ ± 7.96 × 101^a^	–
	benzene, butyl-	104-51-8	C_10_H_14_	1298.80	1029.71	3.54 × 10^2^ ± 8.97 × 101^a^	2.30 × 102 ± 2.16 × 101^b^	1.57 × 102 ± 3.07^c^	1.79 × 102 ± 1.08 × 101^c^	1.94 × 102 ± 1.12 × 101^b^	3.58 × 102 ± 6.99^a^	–
	Piperazine	110-85-0	C_4_H_10_N_2_	1384.50	1286.83	1.8 × 7 × 102 ± 2.08 × 101^b^10^1^	1.75 × 102 ± 1.36 × 10^1b^	1.810^2^7 × 10^2^ ± 1.32 × 10^1b^	2.03 × 10^2^ ± 1.09 × 10^1b^	1.97 × 102 ± 6.50^b^	4.38 × 102 ± 1.40 × 10^1a^	–
	2,3-dimethylpyrazine	5910-89-4	C_6_H_8_N_2_	1349.30	1181.30	1.83 × 10^2^ ± 4.90 × 10^1b^	2.49 × 10^2^ ± 9.91^b^	2.56 × 10^2^ ± 2.78 × 10^1b^	2.48 × 10^2^ ± 6.74^b^	2.21 × 10^2^ ± 1.17 × 10^1b^	8.91 × 10^2^ ± 3.86 × 10^1a^	–
	2,5-dimethylpyrazine	123-32-0	C_6_H_8_N_2_	1328.00	1117.37	3.01 × 10^2^ ± 8.38 × 10^1b^	4.01 × 10^2^ ± 2.96 × 10^1b^	4.01 × 10^2^ ± 2.21 × 10^1b^	3.90 × 10^2^ ± 2.51 × 10^1vb^	3.74 × 10^2^ ± 1.20 × 10^1b^	1.67 × 10^3^ ± 9.15 × 10^1a^	–
Other	alpha -pinene	80-56-8	C_10_H_16_	998.50	346.19	5.96 × 10^2^ ± 1.33 × 10^2b^	9.88 × 10^2^ ± 3.46 × 10^1a^	8.56 × 10^2^ ± 1.80 × 10^1a^	8.83 × 10^2^ ± 9.33^a^	8.58 × 10^2^ ± 3.22 × 10^1a^	5.12 × 10^2^ ± 1.65 × 10^1b^	–
	1-octene ^*^	111-66-0	C_8_H_16_	876.50	274.49	1.68 × 10^2^ ± 2.43 × 10^1c^	2.60 × 10^2^ ± 9.55 × 10^1b^	3.18 × 10^2^ ± 3.38 × 10^1a^	2.20 × 10^2^ ± 6.20 × 10^1b^	1.59 × 10^2^ ± 1.67 × 10^1c^	1.27 × 10^2^ ± 5.72^c^	Gasoline smell
	1,2-dimethoxyethane	110-71-4	C_4_H_10_O_2_	910.30	293.95	1.02 × 10^3^ ± 6.63 × 10^1a^	6.34 × 10^2^ ± 2.55 × 10^1d^	6.19 × 102 ± 5.75 × 10^1d^	7.72 × 102 ± 1.83 × 10^1c^	8.40 × 102 ± 1.39 × 10^1c^	9.93 × 102 ± 3.76^b^	–
	p-methyl anisole	104-93-8	C_8_H_10_O	1438.60	1448.87	4.05 × 102 ± 7.12 × 10^1b^	4.64 × 102 ± 4.04 × 10^1b^	4.55 × 102 ± 2.72 × 10^1b^	4.38 × 102 ± 1.46 × 10^1b^	4.37 × 102 ± 9.92^b^	5.85 × 102 ± 4.64 × 10^1a^	–
	dimethyl disulfide	624-92-0	C_2_H_6_S_2_	1076.00	440.77	2.97 × 10^1^ ± 3.36^e^	4.60 × 10^1^ ± 2.08^10^-1 ^d^	8.48 × 10^1^ ± 2.91^c^	1.69 × 102 ± 1.09 × 10^1b^	2.83 × 102 ± 1.16 × 10^1a^	1.03 × 102 ± 7.63^c^	–

For the same compound, the letter “M” denotes the monomer and “D” denotes the dimer. For each compound, different superscript letters following the SD values indicate significant differences. “^*^” Key volatile organic compounds (VIP > 1). Threshold and description of volatile substances from the Vcf - online system (Accessed on 25 December 2025, http://www.vcf-online.nl/VcfHome.cfm) (4).

**Figure 3:**
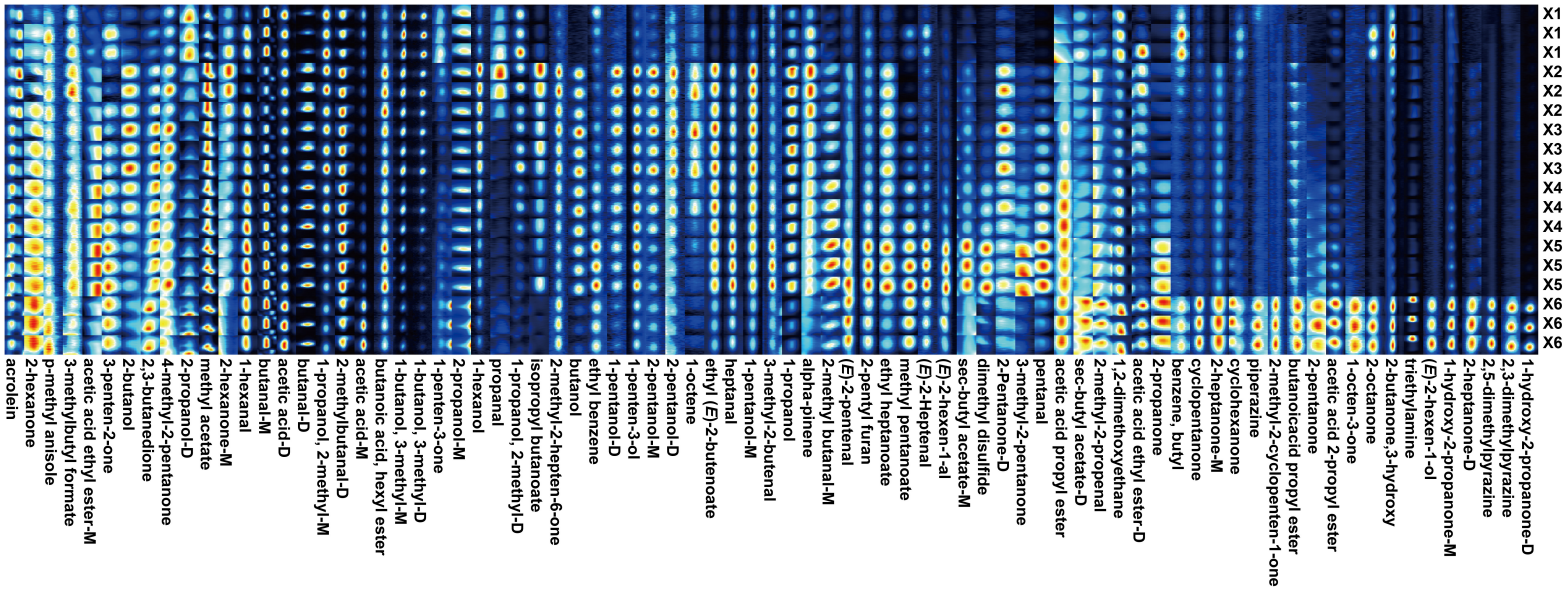
Fingerprint spectra of volatile components in Tartary buckwheat tea during the roasting process. Note: each row represents a sample and each column represents an identified compound, with the brightness of the spot corresponding to the signal intensity.

Volatile components of Tartary buckwheat tea at different roasting stages were analyzed using the GC-IMS system combined with its built-in Gallery Plot plug-in. As shown in the GC-IMS fingerprint in Figure 3, a total of 70 peak signals corresponding to volatile compounds were detected and identified in samples from six roasting stages, indicating a relatively rich composition of flavor substances across the samples. Table 2 lists the volatile flavor compounds identified in each sample, including 12 alcohols, 9 aldehydes, 1 acid, 11 esters, 17 ketones, 2 sulfur-containing compounds, 10 heterocyclic compounds, 7 alkenes, and 1 ether (including monomers and dimers). To further identify and compare changes in volatile flavor substances during Tartary buckwheat tea roasting, characteristic peaks from the GC-IMS fingerprints of samples at each roasting stage (X1–X6) were extracted using the Gallery Plot plug-in, and a fingerprint map of target compounds was constructed (Figure 3). As shown in the figure, the overall signals of volatile compounds were weak at the initial roasting stage (X1), indicating that flavor substances had not yet been fully released. As the roasting process progressed, the signal intensities of key flavor compounds, including nonanal, 2-butanone, and furan, increased markedly from stage X2 to X5 and reached a maximum at stage X4, indicating that this stage is critical for the release and transformation of flavor components. At X6, the signals of some compounds began to decrease, which may be attributable to volatilization losses or degradation reactions induced by prolonged high-temperature exposure. For example, acetic acid and methyl propanal showed strong signals in the mid-stage samples X3–X5, which may be associated with Maillard reactions of carbohydrates or lipid oxidation pathways. In contrast, some aldehydes (hexanal and nonanal) accumulated continuously and may contribute to roasted notes.

### 3.3 PLS-DA model analysis of volatile organic compounds during Tartary buckwheat roasting

**Figure 4:**
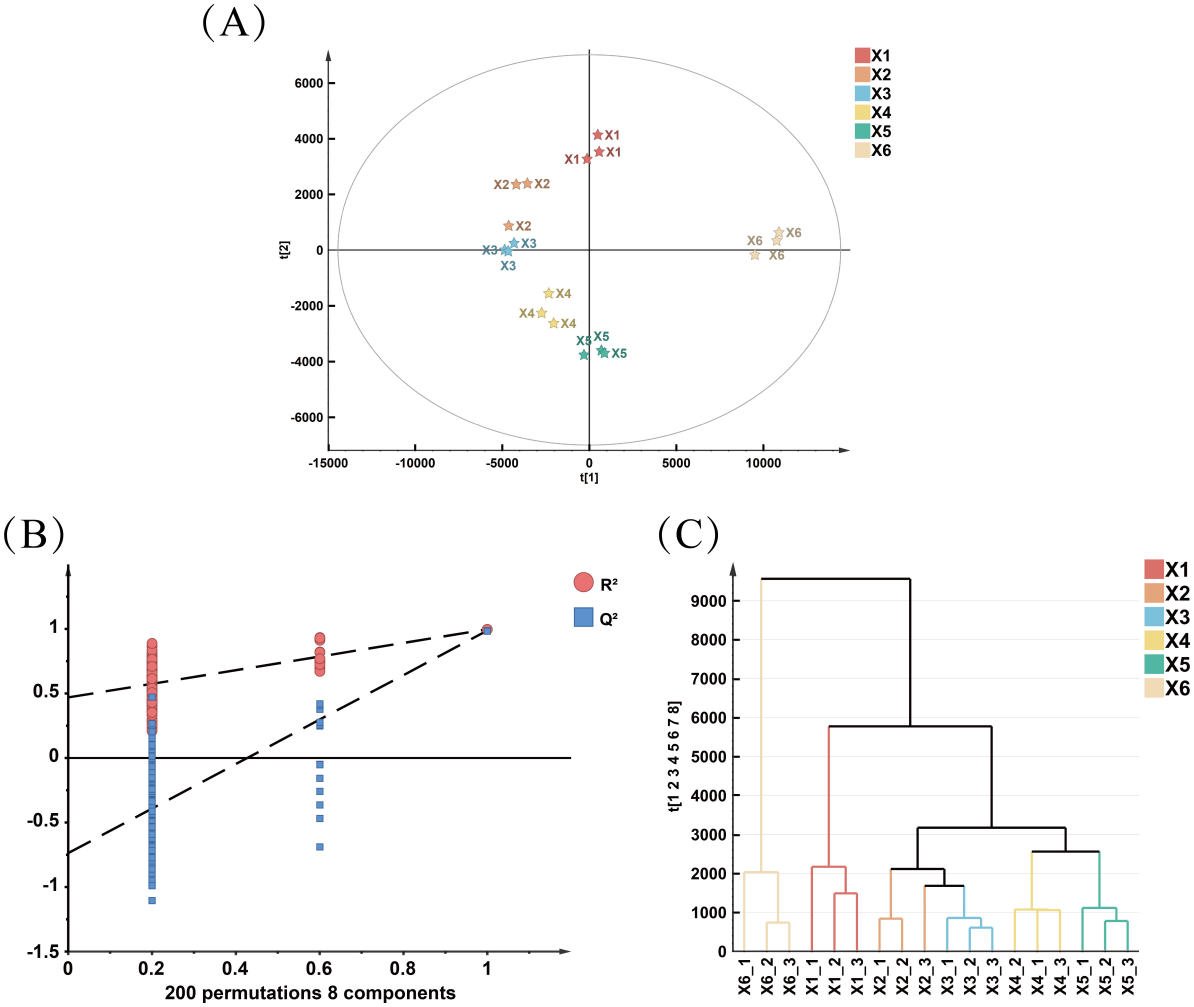
Partial least squares discriminant analysis of volatile compounds in Tartary buckwheat tea during the roasting process **(A)** PLS-DA, **(B)** Permutation test of the PLS-DA model based on 200 permutations, **(C)** Cluster analysis.

To further investigate differences in volatile flavor compounds among samples collected at various time points during Tartary buckwheat tea roasting (X1–X6), dimensionality reduction was performed using a PLS-DA model and hierarchical cluster analysis. PLS-DA is a supervised discriminant statistical method that reduces the dimensionality of complex experimental data, facilitating visualization, discrimination, and prediction (26). In this model, it is essential to determine the explained variance (R^2^X and R^2^Y), Q^2^, and the number of principal components (PCs) required for cross-validated predictive performance (27). As shown in Figure 4A, in this study, R^2^X = 0.997, cumulative R^2^Y = 0.967, and Q^2^ = 0.872. When the difference between R^2^X and R^2^Y is less than 0.1, it indicates high model reliability. Here, the model adequately explained the significant variance in the dataset, achieved good discrimination, and comprehensively captured the features among Tartary buckwheat samples (26). In the PLS-DA plot, sample X1 is clearly located in the upper left quadrant, whereas samples X2 through X6 gradually shift toward the lower quadrants, indicating that the volatile composition changes markedly with increasing roasting time. Notably, samples from stage X4 cluster near the center of the PLS-DA plot, implying pronounced accumulation of flavor substances at this stage during roasting. The distribution pattern in the PLS-DA plot effectively distinguishes differences between pre- and post-roasting samples and reveals a clear temporal progression in flavor composition across the time points. In addition, to verify the reliability of the PLS-DA model and assess the risk of overfitting, 200 permutation tests were performed. As shown in Figure 4B, the intercept of the Q2 regression line was −0.413, which was less than 0, indicating that the model was not obviously overfitted. The intercept of the R^2^ regression line was 0.507. Taken together, the R^2^X, R^2^Y, Q^2^, and permutation test results demonstrated that the PLS-DA model had good stability and reliability in distinguishing Tartary buckwheat tea samples at different roasting stages. The clustering analysis in Figure 4C further confirms the sample grouping trend. Hierarchical clustering shows that X1 and X2 form one cluster on the dendrogram, reflecting similarity in their volatile components. In contrast, X3 through X5 cluster tightly in another group, indicating similar chemical characteristics at these stages. Although X6 shows some similarity to X3 through X5 in the dendrogram, its position is slightly separated, suggesting that flavor components tend to stabilize at this stage, possibly due to volatilization losses and degradation of flavor substances. Overall, the key period during roasting is X3 through X5, during which flavor substances accumulate most markedly and the VOC compositions are highly similar among samples, suggesting that flavor changes at this stage result from the synergistic action of a series of chemical reactions. In contrast, stages X1 and X2 mainly reflect the initial formation of flavor substances, whereas X6 represents stabilization of flavor components.

### 3.4 Screening of characteristic differential volatile organic compounds during Tartary buckwheat roasting

**Figure 5:**
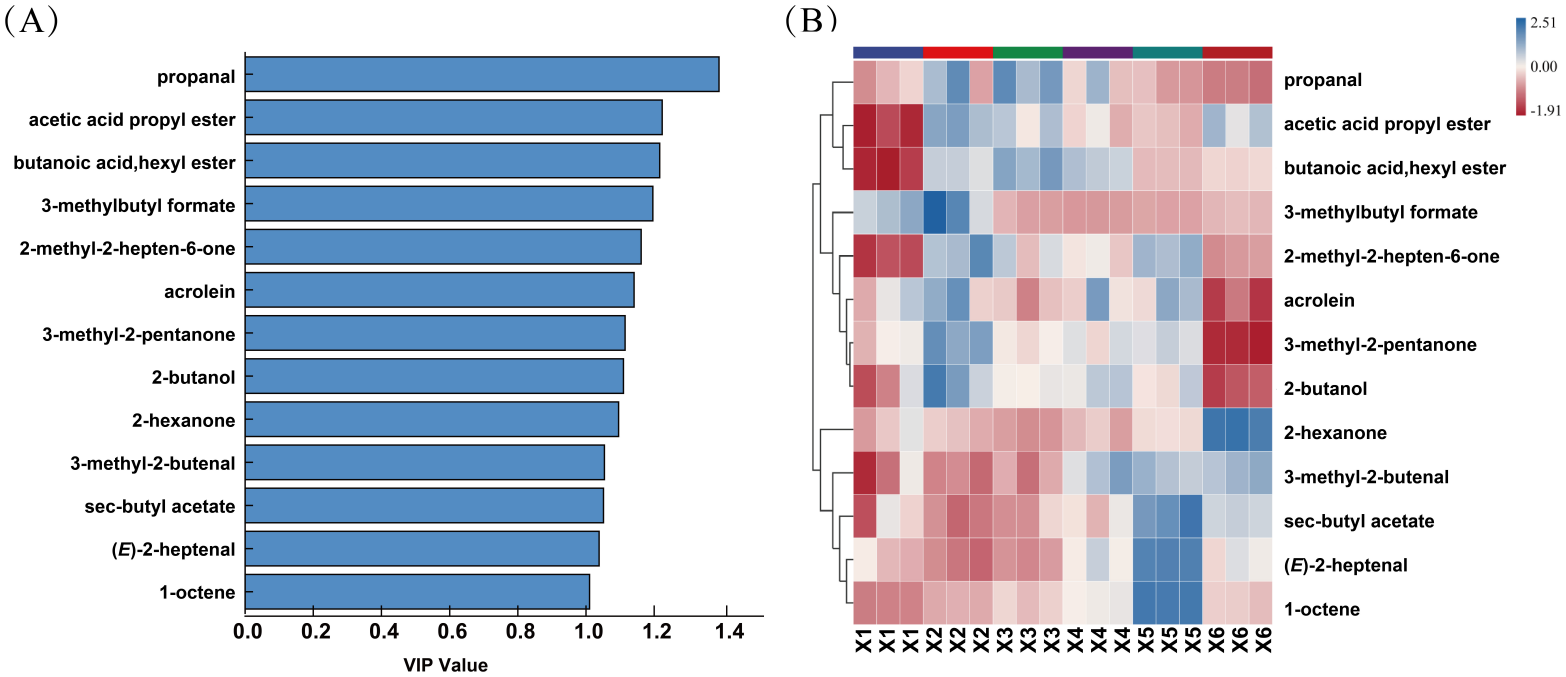
PLS-DA model analysis of volatile compounds during the roasting process of tartary buckwheat **(A)** VIP plot, **(B)** Clustering heatmap.

To identify key discriminant substances during Tartary buckwheat roasting, VIP scores were calculated to evaluate the influence and explanatory power of each variable on sample discrimination (14). A higher VIP value indicates a more pronounced difference, and a VIP score greater than 1 suggests that the corresponding feature compound is highly important for sample classification and discrimination. As shown in Figure 5A, a total of 13 key differential compounds were identified, including four esters (propyl acetate, hexyl butyrate, isoamyl formate, and sec-butyl acetate), four aldehydes (propanal, acrolein, 3-methyl-2-butenal, and (*E*)-2-heptenal), three ketones (methyl heptenone, 3-methyl-2-pentanone, and 2-hexanone), one alcohol (sec-butanol), and one alkene (1-octene). These compounds with VIP > 1 can serve as potential key marker compounds to discriminate different stages during Tartary buckwheat roasting. To further analyze the key discriminant compounds during Tartary buckwheat roasting, cluster analysis was performed based on the compounds with VIP > 1, and a clustering heatmap was generated (Figure 5B). The heatmap links samples from different roasting stages (X1–X6) with peak intensities changes of the key compounds, illustrating the abundances of various flavor substances at each stage. This visualization allows clear identification of the time-dependent migration and differences of each volatile compound during roasting. The heatmap shows that sec-butyl acetate, propyl acetate, (*E*)-2-heptenal, 3-methyl-2-butenal, and 3-methyl-2-pentanone were present at low levels in stages X1–X3 and increased with roasting time, with a marked rise particularly at stage X4. This pattern may be explained by the relatively limited release and formation of volatile flavor compounds during the early roasting stages (X1–X3), when many flavor substances had not yet been fully released or their formation reactions had not become prominent. At stage X4, increased temperature and roasting time promoted the accumulation and release of flavor substances, especially the esters sec-butyl acetate and propyl acetate and the aldehydes (*E*)-2-heptenal and 3-methyl-2-butenal, whose peak intensities rose rapidly. In contrast, sec-butanol, propanal, hexyl butyrate, isoamyl formate, acrolein, and 2-hexanone exhibited a decreasing trend with extended roasting time. This decline may result from further degradation or volatilization of these compounds at high temperatures. Volatile alcohols and aldehydes such as sec-butanol and propanal may decrease during roasting due to pyrolysis or reactions with other components. Esters such as hexyl butyrate and isoamyl formate may undergo decomposition or conversion through reactions with other reactants, leading to a gradual decrease in their peak intensities. Similarly, 2-hexanone may participate in high-temperature reactions, causing its peak intensities to diminish (28). These results indicate that changes in temperature and roasting time significantly impact the formation and transformation of flavor substances, particularly during the X4–X6 stages.

### 3.5 Analysis of characteristic differential volatile organic compounds

**Figure 6:**
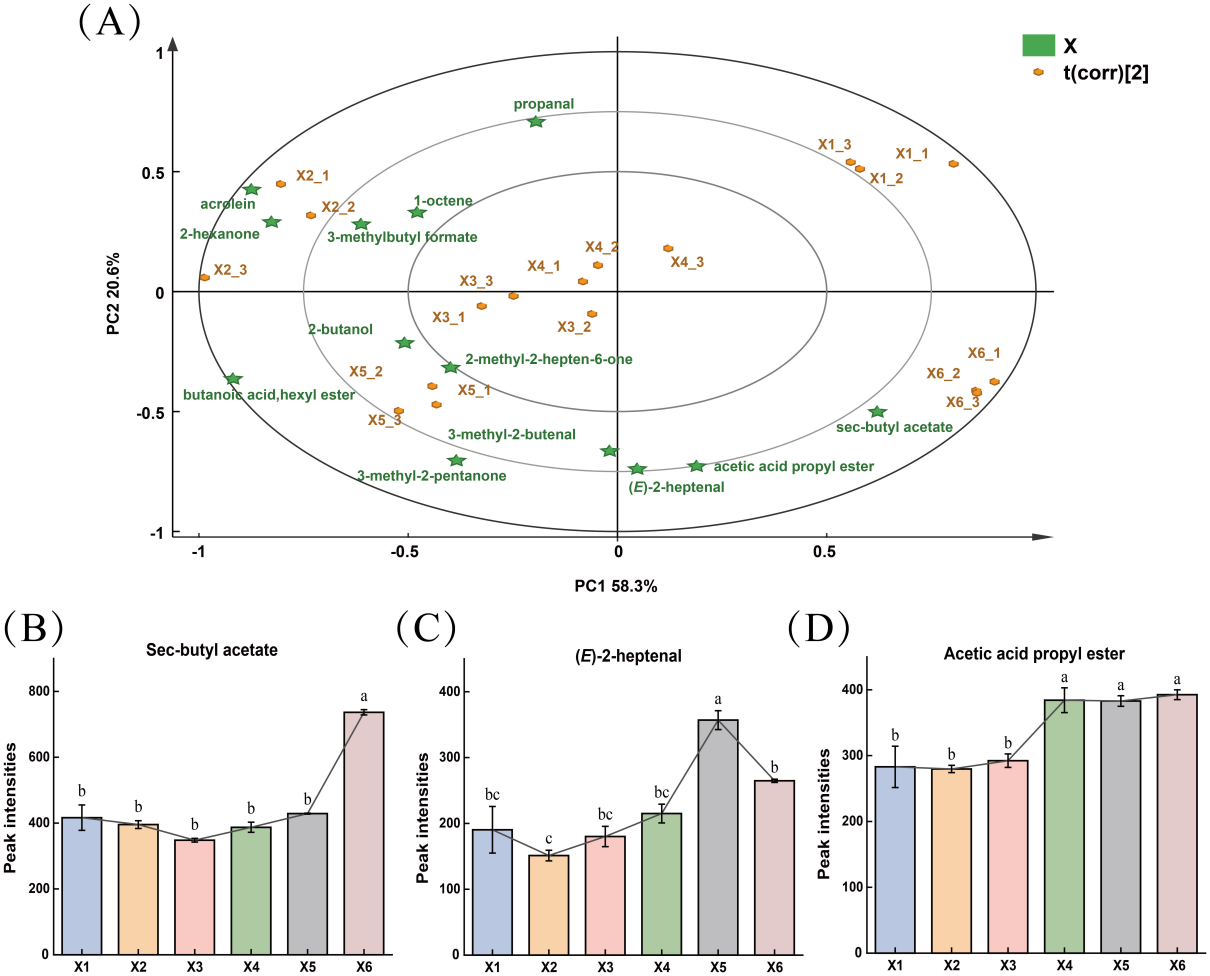
Volatile compound categories of tartary buckwheat during roasting process: **(A)** PLS-DA score plot, **(B)** Peak intensities of sec-butyl acetate, **(C)** Peak intensities of (*E*)-2-heptenal, **(D)** Peak intensities of acetic acid propyl ester.

Further analysis of the PLS-DA loading plot for key volatile components with VIP > 1 reveals the correlations between volatile compounds and samples during Tartary buckwheat roasting (29). As shown in Figure 6A, the x- and y-axes represent the scores of principal components PC1 and PC2, which explain the main directions of variation in volatile components. The relative distance between compounds and sample points reflects differences in the importance of these volatiles during roasting. Compounds closer to a sample point indicate higher associations of their peak intensities or relative abundances with the corresponding stage (30). The results show that propanal, acrolein, 1-octene, 2-hexanone, and isoamyl formate are strongly correlated with the early-stage sample X2. 1-Octene is a product of linoleic acid oxidation, and propanal also belongs to lipid oxidation products. Their appearance marks the release of potato-like aroma, which is a characteristic of the early stage (X2). Tartary buckwheat is rich in unsaturated fatty acids, such as linoleic acid and linolenic acid. In the initial stage of heating, the temperature is not yet enough to trigger intense Maillard reaction to generate pyrazines, but it is enough to make fatty acids undergo oxidative cleavage (11). With extended roasting time, the distributions of X5 and X6 in the loading plot gradually stabilize, indicating that in the late roasting stage, the rates of change in volatile composition and peak intensities slow, particularly regarding the formation and transformation of flavor components. Although the overall trend of change weakens, several key compounds including sec-butanol, hexyl butyrate, methyl heptenone, 3-methyl-2-butenal, 3-methyl-2-pentanone, (*E*)-2-heptenal, propyl acetate, and sec-butyl acetate still show high correlations with X5 and X6. Additionally, the loading plot shows that X6 has the highest positive correlations with sec-butyl acetate, (*E*)-2-heptenal, and propyl acetate. As shown in Figure 6B, the peak intensities of sec-butyl acetate increase markedly across roasting stages, with particularly pronounced changes at X6. This phenomenon is closely related to the formation of volatiles during roasting, as a fatty acid derivative, sec-butyl acetate is typically generated via oxidative decomposition of lipids or esterification reactions, and its fruity and sweet attributes are often described as pineapple-like (31). These reactions generally require relatively high temperatures to proceed appreciably. From the X4 stage, the intensity of sec-butyl acetate increased significantly, and its mechanism can be attributed to the synergistic effect of precursor accumulation and thermal acceleration during medium roasting. In this stage, continuous heat treatment promoted the degradation of unsaturated fatty acids and caramelization of sugars, thereby respectively enriching essential precursors like sec-butyl acetate from lipid oxidation and acetic acid from sugar degradation. These higher temperatures provided the conditions needed to drive the esterification reaction between accumulated alcohols and acids, and in this environment the rate of ester formation exceeded the rate of volatilization, thereby significantly enhancing the fruity and sweet notes in the forming flavor profile (26). Figure 6C shows that the peak intensities of (*E*)-2-heptenal are low at X1-X4, starting from X4, it gradually increases, rises sharply to the highest peak at X5, and then slightly decreases at X6. Although the peak intensities at X6 is lower than those at X5, they remains markedly higher than those at X1–X4, indicating that roasting strongly promotes the accumulation of this compound. Reportedly, its flavor is described as having a green, oily note, and its increased peak intensities is closely related to pyrolysis, esterification, and Maillard reactions during roasting (32). Figure 6D shows that the peak intensities of propyl acetate remain relatively stable from X1 to X3, with no statistically significant difference between X2 and X3 (*p* > 0.05). From X3 to X4, propyl acetate increases gradually and remains at a high level in X4, X5, and X6. In particular, its peak intensities tend to plateau during X4–X6, indicating that late-stage roasting significantly promotes the formation of propyl acetate. Notably, propyl acetate has a mild fruity aroma, which may positively contribute to sensory quality (33).

## 4 Conclusion

In this study, HS-GC-IMS coupled with chemometric analysis was employed to investigate the migration regularity of volatile flavor components in Tartary buckwheat tea during roasting. This study demonstrated that roasting plays a critical role in shaping both the nutritional characteristics and volatile flavor profile of Tartary buckwheat tea. The changes in volatile compounds were strongly stage dependent, indicating that flavor development during roasting is a dynamic process. The X3–X5 roasting stage is critical for aroma development, as volatiles become more distinct and flavor compounds accumulate significantly. From a processing perspective, these findings suggest that precise control of roasting time is essential for balancing nutrient retention and desirable aroma formation. The potential volatile markers identified by HS-GC-IMS combined with chemometric analysis may be useful for distinguishing roasting degrees and monitoring product quality (VIP > 1). This analytical strategy provides a practical approach for process optimization, quality control, and standardized production of Tartary buckwheat tea. Future research should integrate sensory evaluation, aroma activity analysis, and non-volatile metabolomics to further clarify the relationship between precursor transformation, volatile formation, and perceived aroma quality.

## Data Availability

The original contributions presented in the study are included in the article/supplementary material, further inquiries can be directed to the corresponding author.
